# Spatial-temporal epidemiological characteristics of thyroid cancer mortality in China cancer registration areas: analysis based on multiple models

**DOI:** 10.3389/fonc.2026.1858599

**Published:** 2026-09-03

**Authors:** Jiaqi Yang, Zhenhao Zheng, Jianjiang Shao, Yueze Gong, Yu Zhang, Han Wang, Yunhua Hu, Hao Liu, Wen Liu, Yizhong Yan

**Affiliations:** 1 Shihezi University School of Public Health, Shihezi, Xinjiang, China; 2 Department of Ultrasound, the First Affiliated Hospital of Shihezi University, Shihezi, Xinjiang, China; 3 Shihezi University School of Clinical Medical, Shihezi, Xinjiang, China; 4 Key Laboratory for Prevention and Control of Crucial Emerging Infectious Diseases and Public Health Security of The Xinjiang Production and Construction Corps, Shihezi, Xinjiang, China; 5 Key Laboratory of Preventive Medicine, Shihezi University, Shihezi, Xinjiang, China; 6 Key Laboratory of Xinjiang Endemic and Ethnic Diseases (Ministry of Education), School of Medicine, Shihezi University, Shihezi, Xinjiang, China; 7 Department of Gastroenterology, the First Affiliated Hospital of Shihezi University, Shihezi, Xinjiang, China

**Keywords:** cancer registry, death characteristics, prediction analysis, spatial autocorrelation, thyroid cancer

## Abstract

**Objective:**

This study analyzed spatial and temporal patterns of thyroid cancer mortality in China cancer registration areas and predicted future trends, thereby providing a scientific basis for the formulation of prevention and control strategies for thyroid cancer.

**Methods:**

Thyroid cancer death data from 2005 to 2018 were obtained from the China Cancer Registry Annual Report from 2008 to 2021. Joinpoint regression model was used to describe the temporal trends. Age-period-cohort model was used to analyze the age, period and cohort effects on thyroid cancer mortality. The Bayesian age-period-cohort model was used to predict the age-standardized mortality rate (ASMR) of thyroid cancer. Spatial autocorrelation was used to analyze the spatial distribution characteristics of thyroid cancer mortality and identify high-risk areas.

**Results:**

The ASMR of thyroid cancer in China cancer registration areas from 2005 to 2018 was 0.42 per 100,000 and exhibited an increasing trend, with an average annual percent change of 2.1% (95% CI: 1.1%, 3.2%). The upward trend in urban areas and among females was more pronounced than that in rural areas and among males. In terms of age effects, thyroid cancer mortality risk exhibited a W-shaped pattern in the overall population, as well as in urban and rural areas and among females, whereas males showed a pattern of an initial decline followed by an increase with age. The period effects demonstrated increasing trends in the overall population, urban areas, and among males and females. In contrast, the period effect in rural areas initially decreased and subsequently increased, with a turning point in 2010. The cohort effects generally showed a decreasing–increasing–decreasing pattern across the overall population, urban and rural areas, and among males and females. From 2019 to 2030, the ASMR of thyroid cancer in China cancer registration areas will continue to rise. The thyroid cancer mortality showed spatial aggregation, of which Xinjiang, Tibet, and Jilin were high-high aggregation areas.

**Conclusion:**

Thyroid cancer mortality in China cancer registration areas showed an increasing trend from 2005 to 2018, and it will continue to rise in 2019–2030. Attention to thyroid cancer should not be relaxed, especially in Xinjiang, Tibet, and Jilin.

## 1 Introduction

Among endocrine system cancers, thyroid cancer is the most common type. With the continuous improvement of detection techniques and the continuous optimization of treatment strategies, the prognosis of thyroid cancer is generally favorable, with a 5-year survival rate of 84.3% in China (1). However, with the continuous increase in the incidence of thyroid cancer (2), thyroid cancer mortality and the associated disease burden cannot be ignored. According to data from the International Agency for Research on Cancer (IARC), there were approximately 48,000 thyroid cancer deaths worldwide in 2022, representing an increase of 18.77% compared to 2012 (3, 4). In China, the burden of thyroid cancer is equally severe. The Global Burden of Disease (GBD) Study data also showed that there were 7,692 thyroid cancer deaths in China in 2021, an increase of 113.73% compared to 1990. Furthermore, disability-adjusted life years (DALYs) due to thyroid cancer also showed an upward trend in China from 1990 to 2021 (5). Although thyroid cancer mortality remains lower than that of many other cancers, its continued increase remains an important public health issue that urgently needs to be addressed.

Establishing a comprehensive prevention system, optimizing early screening programs, and formulating perfect management strategies throughout the entire process have become extremely crucial (6). To achieve this goal, it is crucial to have a comprehensive understanding of the epidemic characteristics of the disease. In this study, based on the thyroid cancer mortality data from the China Cancer Registry Annual Report, we constructed multiple models to analyze its changing trends, as well as the age-period-cohort effects, and conducted predictive analyses. At the same time, we used spatial autocorrelation methods to analyze its spatial distribution characteristics. These analyses aimed to reveal the distribution patterns and epidemiological trends of thyroid cancer in China cancer registration areas in terms of age, region, and time, and to provide a solid basis for clinical practice and public health decision-making.

## 2 Materials and methods

### 2.1 Data sources

The thyroid cancer data analyzed in this study for the period 2005–2018 were obtained from the China Cancer Registry Annual Report published between 2008 and 2021 (7–20). As provincial-level thyroid cancer data were only available in the China Cancer Registry Annual Report from 2013 to 2021, the spatial analysis in this study was restricted to the period of 2010–2016. The age-standardized mortality rate (ASMR) was calculated based on the Sixth National Population Census published by the National Bureau of Statistics of China in 2010 (21). Thyroid cancer cases were identified using the International Classification of Diseases, 10th Revision (ICD-10/C73) (22). The urban-rural classification was based on the codes for the administrative divisions of the People’s Republic of China (GB/T2260-2007), which categorize cities at or above the prefecture level as urban areas and counties or county-level cities as rural areas.

The data from the China Cancer Registry Annual Report, covering 31 provinces (autonomous regions and municipalities directly under the Central Government) and the Xinjiang Production and Construction Corps (excluding the Hong Kong Special Administrative Region, Macao Special Administrative Region, and Taiwan Province). Quality control was carried out in each cancer registration area at all stages to ensure the comparability, timeliness, completeness and validity of the data. To ensure the authenticity, stability, and comparability of the data, the National Cancer Center implemented rigorous quality control procedures. Any identified issues, such as missing or inconsistent entries, were returned to the local registries for verification and correction.

### 2.2 Joinpoint regression model

The trend of thyroid cancer mortality was described using the annual percentage change (APC), average annual percentage change (AAPC), and their 95% confidence intervals (95% CI) (23). The model partitions the study period into distinct segments and performs trend fitting and optimization independently within each segment. The Monte Carlo permutation test (significance level α = 0.05) was used to determine the number of joinpoints, their locations, and associated *P*-values. The Joinpoint regression model has two forms: the linear model and the log-linear model. The log-linear model is generally chosen when analyzing population-based trends in cancer mortality (23–25):


$$\text{E[y}/\text{x]}={\text{e}}^{{\text{β}}_{0}+{\text{β}}_{1}+{\text{δ}}_{1}{\left(\text{x}-{\text{τ}}_{1}\right)}^{+}+\cdots +{\text{δ}}_{\text{K}}{\left(\text{x}-{\text{τ}}_{\text{K}}\right)}^{+}}$$

Where, e is the base of the natural logarithm, k is the number of turning points, $\tau_{k}$ is the number of unknown turning points, *β*_0_ is the invariant parameter, *β*_1_ is the regression coefficient, *δ_k_* indicates the slope of the k-th segmented function. When ( $\text{x}-\tau_{k}$)>0, ${\left(\text{x}-\tau_{1}\right)}^{+}$ = $\text{x}-{\text{τ}}_{\text{k}}$, otherwise ${\left(\text{x}-\tau_{1}\right)}^{+}$=0.


$$\text{APC}=({\text{e}}^{{\text{β}}_{1}}-1)\times 100$$

$$\text{AAPC}=({\text{e}}^{\sum {\text{ω}}_{i}{\text{β}}_{i}/\sum {\text{ω}}_{i}}-1)\times 100$$

Where, *β*_1_ is the regression coefficient, *β_i_* is the regression coefficient corresponding to each interval, *ω_i_* is the width of the interval span of each segmentation function. When no turning point exists, APC = AAPC. An upward trend is defined as APC > 0 or AAPC > 0 with 95% CI> 0, a downward trend requires APC < 0 or AAPC < 0 with 95% CI< 0. A value equal to 0 indicates no obvious trend.

### 2.3 Age-period-cohort model

This study used the age-period-cohort (APC) model to evaluate the independent effects of age, period, and birth cohort on thyroid cancer mortality (26). The age effect reflects differences in mortality risk across age groups, the period effect reflects temporal changes affecting all age groups simultaneously, and the cohort effect reflects differences in mortality risk among individuals born in different years.

Because age, period, and cohort are linearly dependent (age=period-cohort), the APC model has an inherent identifiability problem. Therefore, the Intrinsic Estimator (IE) was adopted for parameter estimation. It addresses the identifiability problem through its intrinsic parameterization and allows estimation of the age, period, and cohort parameters without imposing user-specified constraints. The formula is expressed as (27–29):


$$\text{Y}=\text{log}(\text{M})=\text{μ}+{\text{αage}}_{1}+{\text{βperiod}}_{1}+{\text{γcohort}}_{1}+\text{ϵ}$$

Where, M represents the mortality, α represents the coefficients for age, β represents the coefficients for period, γ represents the coefficients for cohort, μ is the intercept; ϵ is the random error.

### 2.4 Bayesian age-period-cohort model

The Bayesian age-period-cohort (BAPC) model was applied to predict future trends in thyroid cancer mortality. Compared with the conventional APC model, the BAPC model incorporates Bayesian smoothing priors to estimate the age, period, and cohort effects (30, 31), thereby producing smooth estimates of the temporal effects and alleviating the identifiability problem arising from the exact linear dependency among age, period, and cohort.

Let *Yap* denote the observed number of thyroid cancer deaths in age group *a* during period *p*, and let *Eap* denote the corresponding population at risk. The observed number of deaths was assumed to follow a Poisson distribution:


$${\text{Y}}_{\text{ap}}∼\text{Poisson}({\text{E}}_{\text{ap}}{\text{λ}}_{\text{ap}})$$

where λap is the mortality rate for the corresponding age-period group. A logarithmic link function was specified as:


$$\text{log}({\text{λ}}_{\text{ap}})=\text{μ}+{\text{α}}_{\text{a}}+{\text{β}}_{\text{p}}+{\text{γ}}_{\text{c}}$$

Where  μ is the intercept, and ${\text{α}}_{\text{a}}$, ${\text{β}}_{\text{p}}$, and ${\text{γ}}_{\text{c}}$ represent the age, period, and cohort effects, respectively, with the cohort index defined as c=p−a.

The age, period, and cohort effects were modelled using second-order random walk (RW2) priors. The corresponding precision hyperparameters were assigned the default log-Gamma prior distributions implemented in the BAPC package. In addition, an independent and identically distributed random effect was included to account for extra-Poisson variation.

Posterior inference was performed using the Integrated Nested Laplace Approximation (INLA) algorithm, which provides efficient approximations to the posterior distributions of latent Gaussian models (32). The BAPC model was fitted using the observed mortality and population data from 2005 to 2018. Future mortality was predicted from the posterior distributions of the estimated age, period, and cohort effects under RW2 priors, without introducing an additional drift component or deterministic extrapolation. Using 5-year age groups and annual mortality data, future mortality was predicted for 12 future periods, from 2019 to 2030.

Model predictive performance was assessed using the mean absolute error (MAE), mean squared error (MSE), and mean absolute percentage error (MAPE), with lower values indicating better predictive accuracy. Model fit was assessed using the deviance information criterion (DIC), log pseudo marginal likelihood (LPML), CPO failure rate, and Pearson residuals. Lower DIC values, higher LPML values, lower CPO failure rates, and Pearson residuals with a mean close to 0 and a standard deviation close to 1 were considered indicative of better model fit.

As a sensitivity analysis, the period effect was alternatively specified as a first-order random walk (RW1), and predictions for 2025 were additionally performed to evaluate the robustness of the predicted results.

### 2.5 Spatial autocorrelation analysis

Spatial autocorrelation is a measure used to evaluate whether there is interdependence between the attribute values of a variable and different spatial units within the study area. Commonly used spatial autocorrelation indicators include Moran’s I, Geary’s C, and G statistics (33).

In this study, the global Moran’s I index reflects the spatial distribution status of thyroid cancer mortality across China cancer registration areas. The Moran’s I index takes the value range of [-1, 1], with values close to 1 indicating a very strong positive spatial positive correlation, close to 0 indicating a random distribution, and close to -1 indicating a very strong negative spatial correlation.

Local spatial autocorrelation can explore the type of aggregation that exists between spatial regions, mainly with clustered outlier analysis. The local Moran’s I statistic is used to find out whether there is clustering of observations in a localized area, including high-high clustering, low-low clustering, high-low anomalies, and low-high anomalies.

Since only the 2013–2019 annual reports included cancer data from different provinces in China, this study used spatial autocorrelation to analyze the spatial distribution characteristics of thyroid cancer mortality from 2010–2016.

### 2.6 Statistical analysis

The Joinpoint regression analysis was performed using the Joinpoint Regression Program (version 5.0.2) developed by the National Cancer Institute. The APC model was implemented using Stata 18, and the BAPC model was constructed using R software (version 4.4.3) with the BAPC package (version 0.0.37) and the INLA package (version 24.12.11) for Bayesian inference. Spatial distribution characteristics of thyroid cancer mortality in China cancer registration areas were analyzed and visualized using ArcGIS software (version 10.8). Additional data processing and visualization in R were performed using the tidyverse and ggplot2 packages. The statistical significance level was set at two-tailed α = 0.05.

## 3 Results

### 3.1 Thyroid cancer mortality and trends in China cancer registration areas

In China cancer registration areas, a total of 16,532 thyroid cancer deaths were recorded between 2005 and 2018, with 9,734 deaths in urban areas (58.88%) and 6,798 in rural areas (41.12%), and 6,095 among males (36.87%) and 10,437 among females (63.13%). The thyroid cancer mortality in the overall population was 0.56 per 100,000 population, with 0.69 per 100,000 population in urban areas, 0.47 per 100,000 population in rural areas, 0.40 per 100,000 population in males, and 0.71 per 100,000 population in females. The ASMR in the overall population was 0.42 per 100,000, with 0.45 per 100,000 in urban areas, 0.38 per 100,000 in rural areas, 0.32 per 100,000 in males, and 0.52 per 100,000 in females.

**Figure 1:**
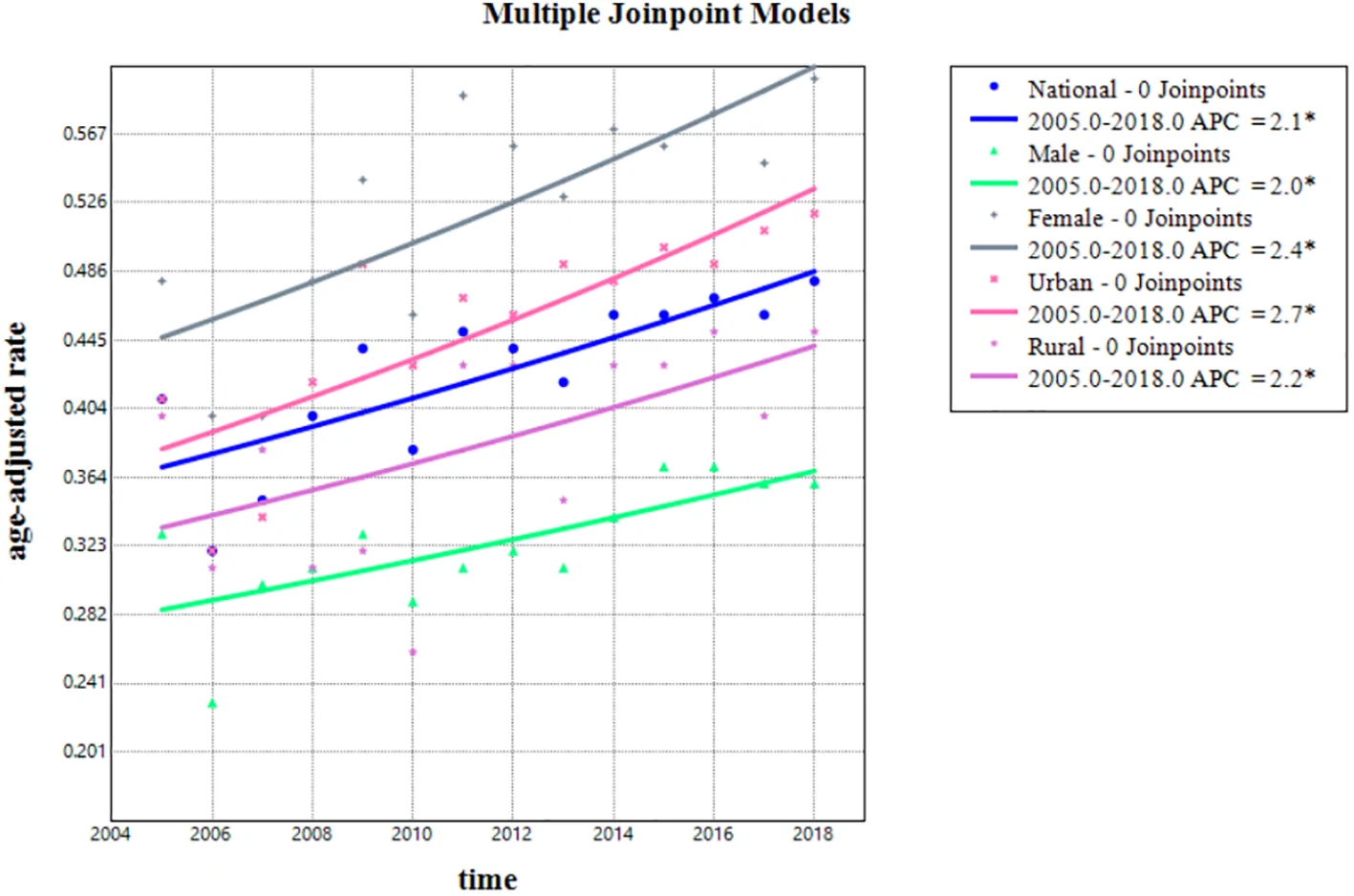
Trends in thyroid cancer mortality in China cancer registration areas, 2005–2018.

From 2005 to 2018, the thyroid cancer ASMR in the overall population of China cancer registration areas increased from 0.41 to 0.48 per 100,000, with an AAPC of 2.1% (95% CI: 1.1%, 3.2%). The AAPCs for urban, rural, male, and female subgroups were 2.7% (95% CI: 1.4%, 3.9%), 2.2% (95% CI: 0.2%, 4.2%), 2.0% (95% CI: 0.7%, 3.2%), and 2.4% (95% CI: 1.1%, 3.7%), respectively (**Tables 1**, **2**, **Figure 1**).

**Table 1:** Mortality of thyroid cancer in China cancer registration areas, 2005-2018 (1/100,000).

Year	Overall			Male			Female			Urban			Rural		
	Deaths	Mortality	ASMR	Deaths	Mortality	ASMR	Deaths	Mortality	ASMR	Deaths	Mortality	ASMR	Deaths	Mortality	ASMR
2005	248	0.45	0.41	91	0.33	0.33	157	0.58	0.48	193	0.47	0.41	55	0.39	0.40
2006	219	0.37	0.32	75	0.25	0.23	144	0.49	0.40	181	0.39	0.32	38	0.29	0.31
2007	247	0.41	0.35	97	0.32	0.30	150	0.51	0.40	192	0.43	0.34	55	0.36	0.38
2008	331	0.50	0.40	120	0.36	0.31	211	0.64	0.48	284	0.54	0.42	47	0.34	0.31
2009	455	0.53	0.44	160	0.37	0.33	295	0.70	0.54	360	0.63	0.49	95	0.34	0.32
2010	546	0.44	0.38	199	0.32	0.29	347	0.56	0.46	423	0.53	0.43	123	0.28	0.26
2011	777	0.53	0.45	254	0.34	0.31	523	0.73	0.59	513	0.59	0.47	264	0.45	0.43
2012	1032	0.52	0.44	364	0.36	0.32	668	0.68	0.56	590	0.59	0.46	442	0.45	0.43
2013	1153	0.51	0.42	410	0.36	0.31	743	0.67	0.53	707	0.63	0.49	446	0.39	0.35
2014	1612	0.56	0.46	587	0.40	0.34	1025	0.72	0.57	905	0.63	0.48	707	0.49	0.43
2015	1865	0.58	0.46	712	0.44	0.37	1153	0.73	0.56	1019	0.66	0.50	846	0.51	0.43
2016	2264	0.59	0.47	860	0.44	0.37	1404	0.75	0.58	1253	0.65	0.49	1011	0.54	0.45
2017	2551	0.58	0.46	979	0.44	0.36	1572	0.73	0.55	1471	0.69	0.51	1080	0.48	0.40
2018	3232	0.62	0.48	1187	0.45	0.36	2045	0.79	0.60	1643	0.70	0.52	1589	0.55	0.45
Total	16532	0.56	0.42	6095	0.40	0.32	10437	0.71	0.52	9734	0.69	0.45	6798	0.47	0.38

ASMR, age-standardized mortality rate.

**Table 2:** Joinpoint Regression analysis results of thyroid cancer mortality rate in China cancer registration areas, 2005-2018 (%).

Index	Overall	Male	Female	Urban	Rural
Year	2005-2018	2005-2018	2005-2018	2005-2018	2005-2018
APC	2.1	2.0	2.4	2.7	2.2
(95% CI)	(1.1, 3.2)	(0.7, 3.2)	(1.1, 3.7)	(1.4, 3.9)	(0.2, 4.2)
AAPC	2.1	2.0	2.4	2.7	2.2
(95% CI)	(1.1, 3.2)	(0.7, 3.2)	(1.1, 3.7)	(1.4, 3.9)	(0.2, 4.2)
AAPC, average annual percentage change; APC, annual percent change					

### 3.2 Age-period-cohort analysis of thyroid cancer mortality in China cancer registration areas

From 2005 to 2018, the age effects on thyroid cancer mortality risk in urban areas, rural areas, and among females in China cancer registration areas were all consistent with the pattern observed in the overall population, with all groups showing a W-shaped curve. For populations over 25 years of age, the thyroid cancer mortality risk increased with age. The two local minima in age effects for urban and rural areas appeared in the 10–14 and 20–24 age groups, whereas the minima for the overall population appeared in the 5–9 and 20–24 age groups. The thyroid cancer mortality risk in males showed a trend of first decreasing and then increasing with age, with the lowest risk in the 20–24 age group.

The period effects in urban areas, males, and females in China cancer registration areas all showed increasing trends from 2005 to 2015, consistent with the overall population. The effect coefficient for the overall population increased from −0.32 to 0.40; that for urban areas rose from −0.48 to 0.47; that for males increased from −0.36 to 0.41; and that for females increased from −0.33 to 0.40. The period effects in rural areas first decreased and then increased, with a turning point observed in 2010.

**Figure 2:**
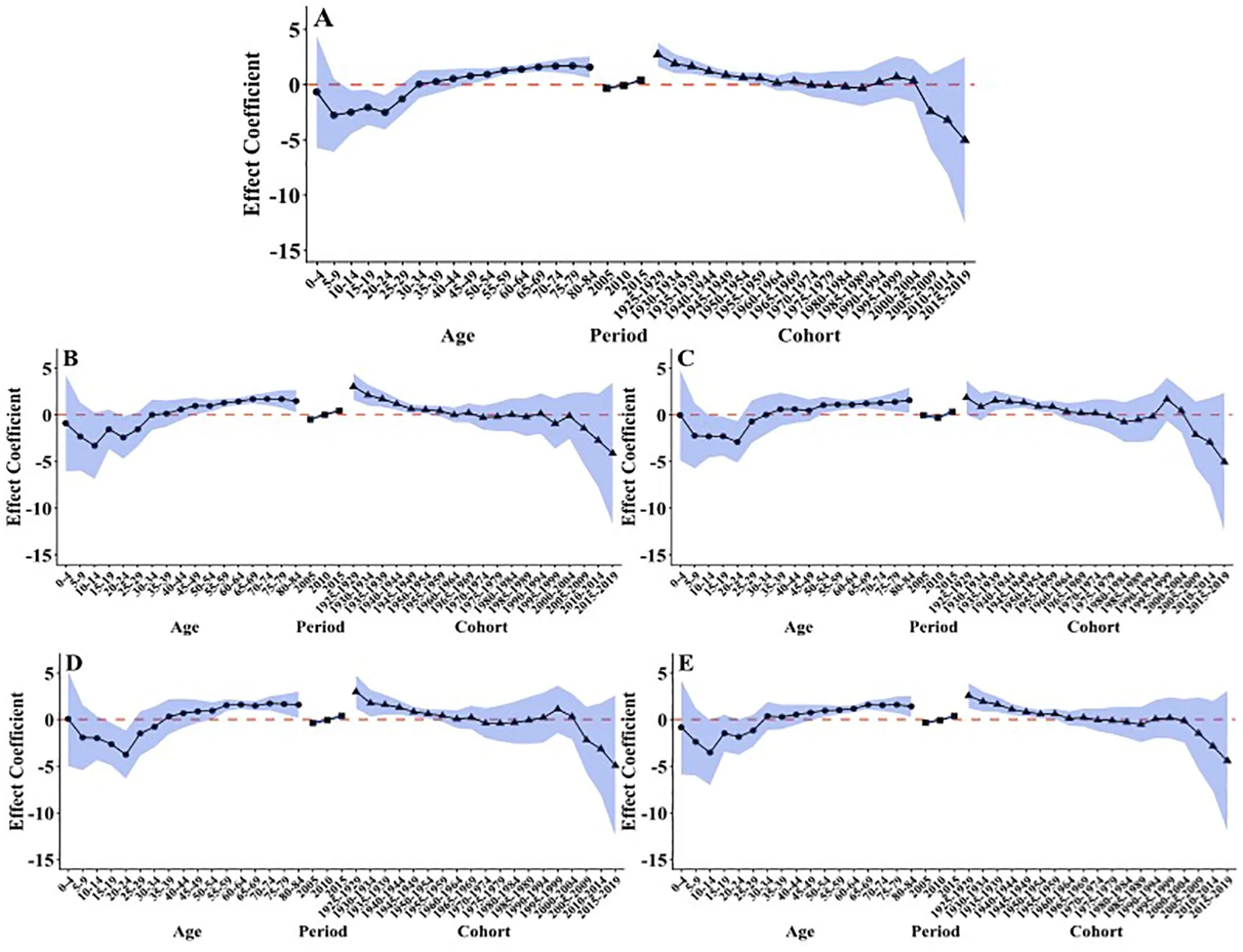
Age-period-cohort analysis of thyroid cancer mortality in overall **(A)**, urban **(B)**, rural **(C)**, male **(D)**, and female **(E)** in China cancer registration areas, 2005–2018.

The cohort effects in the overall population, in urban areas, in rural areas, and among males and females generally showed a decreasing–increasing–decreasing trend. The mortality risk was the highest in the 1925–1929 birth cohort, with effect coefficients of 2.73 for the overall population, 3.03 for urban areas, 1.85 for rural areas, 2.99 for males, and 2.56 for females (**Figure 2**; **Supplementary Table S1**).

### 3.3 Bayesian age-period-cohort model

Separate BAPC models were fitted for the overall population, urban areas, rural areas, males, and females in China cancer registration areas. The models demonstrated good predictive performance, with MAPE values ranging from 6.49% to 8.18% and low MAE and MSE values across all analyses. In addition, the Pearson residuals were centered near 0, with standard deviations close to 1, while low CPO failure rates indicated satisfactory model calibration and predictive stability (**Table 3**).

**Table 3:** Performance metrics of BAPC models.

Dimension	MAE (/10^5^)	MSE (/10^5^)^2^	MAPE (%)	Pearson residual mean ± SD	DIC	LPML	CPO failure rate (%)
Overall	0.038	0.002	8.10	-0.082 ± 0.931	1450.48	-738.53	0.40
Urban	0.042	0.002	8.18	-0.077 ± 0.933	1328.98	-674.97	1.59
Rural	0.028	0.001	6.72	-0.095 ± 0.700	1197.74	-653.99	0.79
Male	0.023	0.001	6.49	-0.068 ± 0.874	1139.76	-573.27	1.19
Female	0.040	0.002	6.96	-0.079 ± 0.923	1334.98	-674.99	0.40

BAPC, Bayesian age-period-cohort; MAE, mean absolute error; MSE, mean squared error; MAPE, mean absolute percentage error; SD, standard deviation; DIC, deviance information criterion; LPML, log pseudo marginal likelihood; CPO, conditional predictive ordinate.

The BAPC model predicts that the ASMR of thyroid cancer in the overall population of China cancer registration areas will continue to increase from 2019 to 2030. The predicted ASMR for 2020, 2025, and 2030 was 0.51/100,000 (95% CI: 0.45, 0.56), 0.60/100,000 (95% CI: 0.40, 0.80), and 0.74/100,000 (95% CI: 0.26, 1.22), respectively.

Across different regions, the ASMR was predicted to increase, with urban areas consistently exhibiting higher rates than rural areas. The urban ASMR was predicted to increase from 0.54/100,000 (95% CI: 0.49, 0.59) in 2019 to 0.88/100,000 (95% CI: 0.13, 1.63) in 2030, while the rural ASMR was predicted to increase from 0.46/100,000 (95% CI: 0.38, 0.54) to 0.67/100,000 (95% CI: 0.16, 1.18) during the same period.

**Figure 3:**
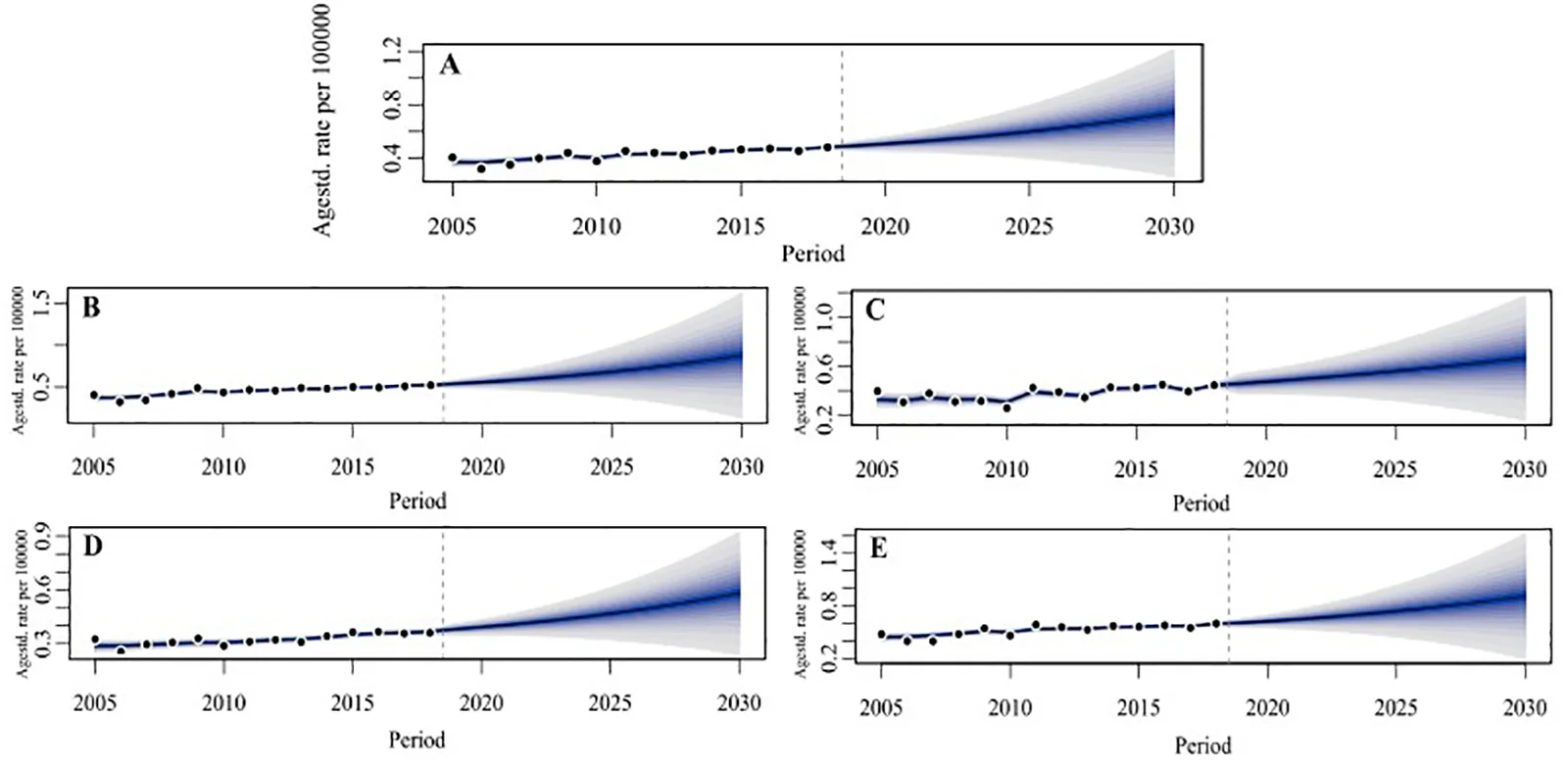
Predicted ASMR for thyroid cancer in overall **(A)**, urban **(B)**, rural **(C)**, male **(D)**, and female **(E)** in China cancer registration areas, 2019–2030.

Regarding gender disparities, the ASMR was predicted to show an upward trend in both males and females, with females consistently exhibiting higher rates than males. The female ASMR was predicted to increase from 0.61/100,000 (95% CI: 0.55, 0.66) in 2019 to 0.91/100,000 (95% CI: 0.20, 1.62) in 2030, whereas the male ASMR was predicted to increase from 0.38/100,000 (95% CI: 0.35, 0.41) to 0.58/100,000 (95% CI: 0.24, 0.92) over the same period (**Table 4**; **Figure 3**).

**Table 4:** Age-standardized mortality of thyroid cancer in China cancer registration areas, 2019–2030 (1/100,000).

Year	Overall		Urban		Rural		Male		Female	
	ASMR	95% CI	ASMR	95% CI	ASMR	95% CI	ASMR	95% CI	ASMR	95% CI
2019	0.49	(0.45,0.53)	0.54	(0.49,0.59)	0.46	(0.38,0.54)	0.38	(0.35,0.41)	0.61	(0.55,0.66)
2020	0.51	(0.45,0.56)	0.56	(0.48,0.63)	0.47	(0.38,0.57)	0.39	(0.35,0.44)	0.62	(0.55,0.70)
2021	0.52	(0.45,0.60)	0.58	(0.47,0.68)	0.49	(0.37,0.61)	0.40	(0.35,0.46)	0.64	(0.53,0.75)
2022	0.54	(0.44,0.64)	0.60	(0.45,0.74)	0.51	(0.37,0.65)	0.42	(0.34,0.49)	0.66	(0.52,0.81)
2023	0.56	(0.43,0.68)	0.62	(0.44,0.81)	0.52	(0.35,0.69)	0.43	(0.34,0.53)	0.69	(0.50,0.87)
2024	0.58	(0.42,0.74)	0.65	(0.41,0.89)	0.54	(0.34,0.74)	0.45	(0.33,0.57)	0.71	(0.47,0.95)
2025	0.60	(0.40,0.80)	0.68	(0.38,0.98)	0.56	(0.32,0.80)	0.47	(0.32,0.61)	0.74	(0.44,1.03)
2026	0.62	(0.38,0.86)	0.71	(0.35,1.08)	0.58	(0.30,0.86)	0.49	(0.31,0.66)	0.77	(0.41,1.13)
2027	0.65	(0.36,0.94)	0.75	(0.31,1.19)	0.60	(0.27,0.93)	0.51	(0.30,0.72)	0.80	(0.37,1.23)
2028	0.68	(0.33,1.02)	0.79	(0.26,1.32)	0.62	(0.24,1.01)	0.53	(0.28,0.78)	0.83	(0.32,1.35)
2029	0.71	(0.30,1.11)	0.83	(0.20,1.47)	0.65	(0.20,1.09)	0.55	(0.26,0.85)	0.87	(0.26,1.48)
2030	0.74	(0.26,1.22)	0.88	(0.13,1.63)	0.67	(0.16,1.18)	0.58	(0.24,0.92)	0.91	(0.20,1.62)

ASMR, age-standardized mortality rate; 95% CI, 95% confidence interval.

Sensitivity analyses were conducted by changing the period effect from RW2 to RW1 and by comparing predictions for 2025 and 2030. The predicted ASMR trends remained highly consistent across the alternative model specifications, supporting the robustness of the BAPC model (**Supplementary Figures S1**, **S2**).

### 3.4 Spatial autocorrelation analysis of thyroid cancer mortality in China cancer registration areas

**Figure 4:**
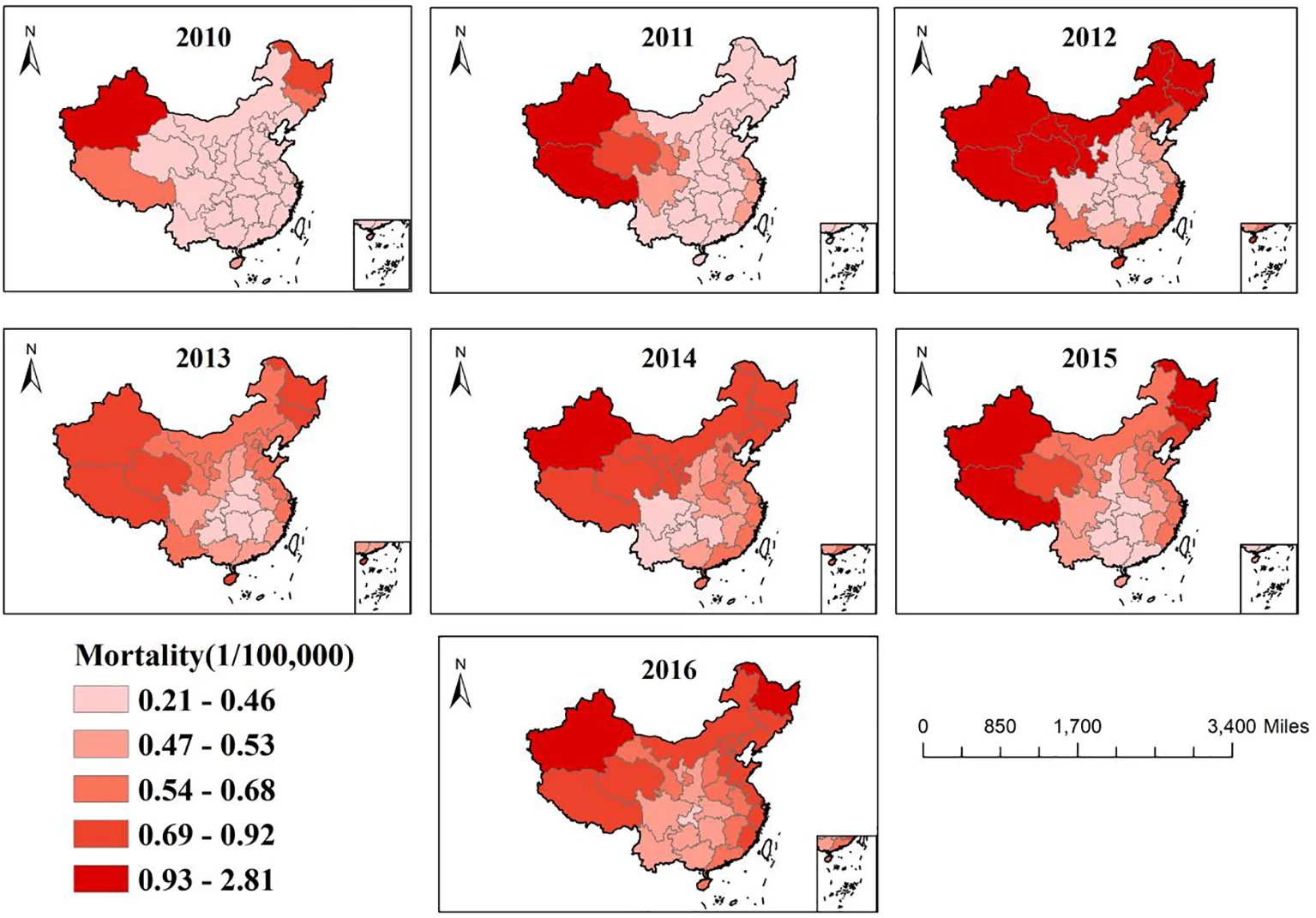
Thyroid cancer mortality in China cancer registration areas, 2010-2016. (Map from the Ministry of Natural Resources of China, cartographic license: GS (2019) 1822). Spatial analyses were conducted only for 2010 -2016 because refined provincial mortality data were available only during this period.

From 2010 to 2016, regions with high thyroid cancer mortality in China cancer registration areas were mainly concentrated in Xinjiang, Inner Mongolia, North China, East China, and Northeast China (**Figure 4**).

Global spatial autocorrelation analysis showed that there was a significant positive spatial autocorrelation of thyroid cancer mortality in China cancer registration areas from 2010 to 2016, indicating spatial clustering (**Table 5**).

**Table 5:** Global Moran’s *I* index of thyroid cancer mortality in China from 2010 to 2016.

Year	Moran’s *I* index	Expectation index	Variance	*Z*	*P*
2010	0.463	-0.03	0.007	6.045	0.000
2011	0.632	-0.03	0.004	10.823	0.000
2012	0.568	-0.03	0.007	7.303	0.000
2013	0.541	-0.03	0.007	6.493	0.000
2014	0.272	-0.03	0.004	5.070	0.000
2015	0.655	-0.03	0.005	10.142	0.000
2016	0.504	-0.03	0.007	6.346	0.000

**Figure 5:**
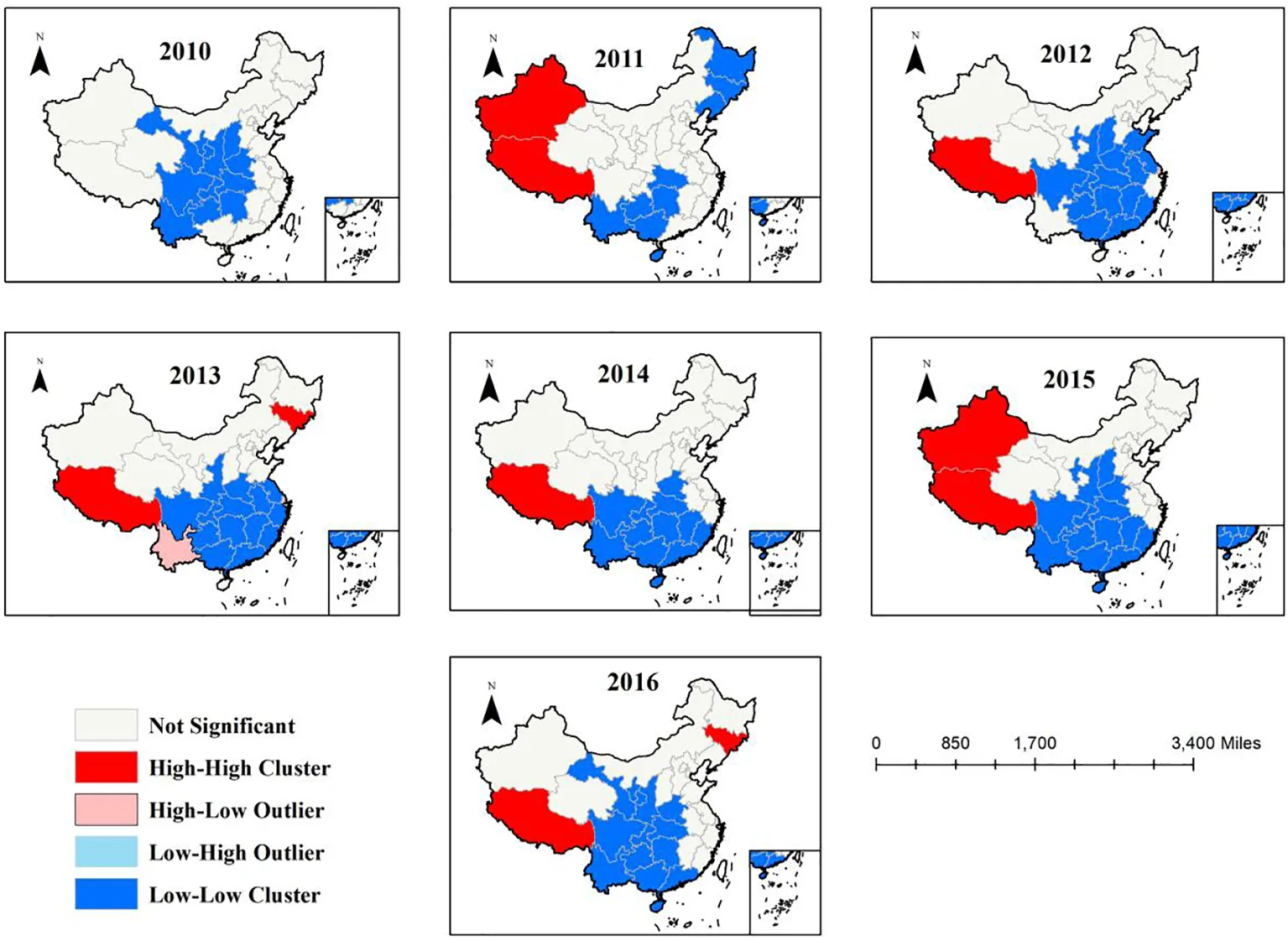
Local spatial autocorrelation of thyroid cancer mortality in China cancer registration areas, 2010-2016. (Map from the Ministry of Natural Resources of China, cartographic license: GS (2019) 1822). Spatial analyses were conducted only for 2010 -2016 because refined provincial mortality data were available only during this period.

To further identify the clustered regions, local spatial autocorrelation analysis was performed on thyroid cancer mortality. The results showed that from 2010 to 2016, there were high-high, low-low, and high-low clustering patterns in some regions. Two high-high clustering regions were identified in 2011. Jilin Province was identified as a new high-high clustering region in 2013 and 2016. From 2013 to 2016, the high-high clustering regions were Xinjiang, Tibet, and Jilin. There were eleven low-low clustering regions in 2010, which extended eastward in 2011 and shifted towards South China from 2012 to 2016, eventually resulting in thirteen low-low clustering regions (**Figure 5**).

## 4 Discussion

The thyroid cancer mortality in China cancer registration areas showed an upward trend from 2005 to 2018, with significant gender and urban-rural disparities. The AAPC was higher in urban areas than rural areas, with urban areas at 2.7% (95% CI: 1.4%, 3.9%) and rural areas at 2.2% (95% CI: 0.2%, 4.2%). The gender disparity indicated a higher AAPC in females at 2.4% (95% CI: 1.1%, 3.7%) and males at 2.0% (95% CI: 0.7%, 3.2%). In addition, among the population aged over 25 years, the mortality risk of thyroid cancer increased with age. The ASMR of thyroid cancer will continue to rise from 2019 to 2030. A high degree of clustering of thyroid cancer deaths was observed in Xinjiang, Tibet, and Jilin. These findings highlight the need for sustained attention to thyroid cancer.

Between 2005 and 2018, both thyroid cancer mortality and ASMR increased in China cancer registration areas. This trend is likely driven by multiple factors. The clinical manifestations of thyroid cancer are often nonspecific. Previous studies have reported that only 38% of patients with thyroid cancer present with clinical symptoms at diagnosis. Among symptomatic patients, 74% initially present with a palpable neck mass, 15% with throat or neck discomfort accompanied by dysphagia, and only 11% with cough, voice changes, dyspnea, or symptoms related to metastatic disease (34). A meta-analysis found significantly lower disease-specific mortality among screen-detected cases compared with those diagnosed after clinical presentation, indicating that delayed detection until symptoms prompt care is associated with increased mortality risk (35). Second, anaplastic thyroid carcinoma (ATC) is characterized by highly aggressive biological behavior, rapid progression, frequent distant metastasis, and an extremely poor prognosis (36, 37). Previous studies have reported an increasing trend in the age-standardized incidence rate of ATC in China (38). This increasing proportion of a highly aggressive subtype may partly contribute to the rising mortality trend observed in our study. In addition, previous studies have shown that the incidence of thyroid cancer in China has continued to increase (39), and the increasing incidence has been reported to be correlated with rising mortality (40).

Between 2005 and 2018, both thyroid cancer mortality and ASMR were higher in females than in males, and the increase in ASMR was greater among females, indicating a marked sex disparity. Previous studies have reported that thyroid cancer incidence is substantially higher in women than in men, with women accounting for approximately three-quarters of newly diagnosed cases (41). This epidemiological pattern is consistent with the higher mortality observed among women in the present study. In addition, from a biological perspective, previous studies have reported that estrogen-activated estrogen receptor-α may mediate the growth and malignant progression of thyroid cancer (42), providing a potential biological explanation for the sex disparities observed in our study.

From 2005 to 2018, both thyroid cancer mortality and ASMR were higher in urban areas than in rural areas, and the increase in ASMR was greater in urban areas, indicating a marked urban-rural disparity. This urban-rural disparity is consistent with previous findings from China (43). Notably, a study based on 1,343 air quality monitoring stations across China from 2013 to 2019 found that areas with higher levels of urbanization generally exhibited higher concentrations of PM_2.5_ and O_3_ (44), suggesting different patterns of air pollutant exposure among urban populations. In addition, previous reviews have indicated that long-term exposure to PM_2.5_, O_3_, and NO_x_ may be associated with an increased risk of thyroid cancer (45). These epidemiological patterns were consistent with the urban-rural differences observed in the present study.

The age effect coefficient of thyroid cancer mortality increased with age among individuals aged over 25 years, reaching its highest level in the 75–84-year age group. Previous studies have shown that ageing is accompanied by a gradual decline in immune function and longer cumulative exposure to environmental carcinogens, such as ionizing radiation (46). In addition, multimorbidity is more prevalent among older adults. These characteristics were consistent with the increasing age effect observed in the present study. The period effect analysis showed an overall increasing trend in mortality risk. Previous studies have reported that improvements in healthcare services and diagnostic technologies during the study period were accompanied by increased detection of thyroid cancer (47). In addition, the proportion of anaplastic thyroid carcinoma among thyroid cancer deaths has also increased (48). However, the period effect in rural areas first decreased and then increased. China launched the New Rural Cooperative Medical Scheme (NCMS) in 2003, and approximately 96% of the rural population was covered by 2010 (49). Previous studies have reported that the expansion of NCMS was accompanied by improvements in healthcare access and increased utilization of outpatient and inpatient services among insured rural residents (50, 51). Although the overall birth cohort effect exhibited a downward trend, thyroid cancer mortality in the overall cancer registration areas showed a localized rebound during 1995–1999, with a significant increase in rural areas. This increase may be related to excessive iodine intake. China fully implemented the universal salt iodization policy in 1994. During the early stage of the policy, the iodine concentration in iodized salt was relatively high, and precise monitoring was limited, particularly in rural areas. Studies have reported that excessive iodine intake was associated with a higher risk of thyroid cancer compared with normal iodine intake (52).

The ASMR of thyroid cancer in China cancer registration areas is predicted to continue increasing between 2019 and 2030. Prediction uncertainty increased with the length of the prediction horizon. Therefore, the predictions should be interpreted primarily as reflecting the overall direction of future mortality trends.

Spatial autocorrelation analysis showed that from 2010 to 2016, thyroid cancer mortality was higher in western and northern China, and in 2011, Xinjiang and Tibet were identified as high-high cluster areas. This phenomenon may be associated with the uneven distribution of medical resources across regions. Medical resources are mainly concentrated in the densely populated and developed eastern regions, whereas the vast and sparsely populated western regions lack medical resources (53). The limited diagnostic and treatment conditions in provinces such as Xinjiang and Tibet may lead to delays in the diagnosis and treatment of thyroid cancer (54). From 2012 to 2016, the high-high cluster areas shifted to Tibet and Jilin. In Tibet, previous studies have reported substantial geographic variations in medical resource accessibility, with some regions experiencing longer travel times to healthcare facilities and limited medical capacity (55). Previous research has also suggested that residents in Tibetan regions may have relatively limited awareness of seeking timely medical care after illness (56), which may influence healthcare-seeking behaviors. Jilin Province newly entered the high–high cluster area in 2013 and 2016, which may be associated with its relatively high degree of population aging. Data from the Seventh National Population Census show that the proportion of the population aged 65 and above in Jilin province reached 15.61%, far exceeding the national average (13.50%) (57), and its aging level ranks among the highest in the country. Elderly patients often have comorbidities, and their tumors tend to be more aggressive and have a poorer prognosis (58). A high proportion of elderly people is likely to increase the risk of cancer incidence or mortality in the region, thereby forming a high-high cluster.

This study has several limitations. First, the data used in this study were obtained from the China Cancer Registry Annual Report, which provides only overall thyroid cancer mortality data and does not include detailed information on pathological subtypes. Therefore, differences in mortality trends among different pathological subtypes could not be further analyzed. Second, due to the inherent approximately three-year reporting lag of the cancer registry system and the continuous expansion of the cancer registry network in recent years, mortality data from the earlier years may deviate from the actual disease burden. Third, the BAPC predictions were generated based on historical age, period, and cohort trends and assumed that these temporal patterns would continue into the future. The model did not account for potential future changes in healthcare policies, diagnostic practices, treatment strategies, or other unforeseen factors that may influence thyroid cancer mortality. Therefore, the predicted results may be subject to bias. Fourth, as an ecological study based on population-level data, this study could not account for individual-level confounding factors. Therefore, the findings should not be interpreted as evidence of causal relationships. Fifth, because data suitable for spatial analysis were only available for the period from 2010 to 2016, the spatial patterns identified in this study may not fully reflect the long-term spatial variation in thyroid cancer mortality. Sixth, although the National Cancer Center has implemented rigorous quality control procedures, regional variations in the completeness and quality of cancer registration may still exist, which could have influenced the observed spatial distribution of thyroid cancer mortality. Finally, advances in diagnostic techniques and changes in clinical diagnostic practices during the study period may also have affected the reported trends in thyroid cancer mortality.

## 5 Conclusion

Thyroid cancer mortality in China cancer registration areas showed an increasing trend from 2005 to 2018, with Xinjiang, Tibet, and Jilin consistently identified as high-high clustering regions of thyroid cancer mortality during this period. From 2019 to 2030, the ASMR of thyroid cancer in China cancer registration areas is predicted to continue increasing. These findings provide important evidence on the temporal trends, spatial distribution, and future burden of thyroid cancer mortality in China and may help inform the development of region-specific public health strategies. Given the persistent increase in thyroid cancer mortality, continued surveillance of thyroid cancer mortality and optimized allocation of healthcare resources may be warranted, particularly in high-risk regions. In areas with persistently high mortality clustering, targeted screening and early detection initiatives may be considered in accordance with local disease burden and healthcare capacity, together with improved access to standardized diagnosis. Such population- and region-specific public health strategies may help reduce regional disparities and strengthen thyroid cancer control in China.

## Data Availability

Publicly available datasets were analyzed in this study. This data can be found here: The data come from the China Cancer Registry Annual Report (2008-2021) issued by China National Cancer Center.
