# Supplementary material for: Spatial-temporal epidemiological characteristics of thyroid cancer mortality in China cancer registration areas: analysis based on multiple models
Source: Front Oncol. 2026 Sep 3;16:1858599. doi: 10.3389/fonc.2026.1858599 (PMC13581727; doi:10.3389/fonc.2026.1858599)
Supplement: Supplementary file 1 [file SupplementaryFile1.docx]

Table S1 Results of the age-period-cohort model analysis of thyroid cancer mortality in China cancer registration areas

| Variables | Overall | | | Urban | | | Rural | | | Male | | | Female | | |
| --- | --- | --- | --- | --- | --- | --- | --- | --- | --- | --- | --- | --- | --- | --- | --- |
|  | Effect Coefficient | 95%CI | SE | Effect Coefficient | 95%CI | SE | Effect Coefficient | 95%CI | SE | Effect Coefficient | 95%CI | SE | Effect Coefficient | 95%CI | SE |
| **Age** |  |  |  |  |  |  |  |  |  |  |  |  |  |  |  |
| 0-4 | -0.66 | (-5.73,4.42) | 2.59 | -0.89 | (-6.07,4.29) | 2.64 | -0.02 | (-4.87,4.82) | 2.47 | 0.08 | (-5.00,5.15) | 2.59 | -0.83 | (-5.87,4.21) | 2.57 |
| 5-9 | -2.77 | (-6.09,0.55) | 1.69 | -2.31 | (-5.96,1.34) | 1.86 | -2.22 | (-5.74,1.31) | 1.80 | -1.91 | (-5.43,1.61) | 1.80 | -2.38 | (-6.00,1.24) | 1.85 |
| 10-14 | -2.50 | (-4.47,-0.53) | 1.00 | -3.30 | (-6.88,0.27) | 1.82 | -2.30 | (-4.56,-0.03) | 1.16 | -1.98 | (-4.35,0.38) | 1.21 | -3.54 | (-7.04,-0.04) | 1.79 |
| 15-19 | -2.06 | (-3.65,-0.47) | 0.81 | -1.54 | (-3.65,0.58) | 1.08 | -2.29 | (-4.39,-0.18) | 1.07 | -2.63 | (-4.91,-0.36) | 1.16 | -1.46 | (-3.45,0.52) | 1.01 |
| 20-24 | -2.52 | (-4.07,-0.96) | 0.79 | -2.43 | (-4.71,-0.14) | 1.17 | -2.90 | (-5.13,-0.68) | 1.14 | -3.75 | (-6.34,-1.17) | 1.32 | -1.86 | (-3.77,0.05) | 0.97 |
| 25-29 | -1.30 | (-2.70,0.10) | 0.72 | -1.53 | (-3.36,0.30) | 0.93 | -0.72 | (-3.00,1.57) | 1.16 | -1.49 | (-3.86,0.88) | 1.21 | -1.18 | (-2.89,0.54) | 0.87 |
| 30-34 | 0.05 | (-1.21,1.30) | 0.64 | 0.01 | (-1.62,1.64) | 0.83 | 0.02 | (-2.07,2.10) | 1.06 | -0.77 | (-2.95,1.41) | 1.11 | 0.38 | (-1.14,1.91) | 0.78 |
| 35-39 | 0.28 | (-0.79,1.35) | 0.55 | 0.14 | (-1.25,1.54) | 0.71 | 0.61 | (-1.18,2.39) | 0.91 | 0.33 | (-1.55,2.21) | 0.96 | 0.28 | (-1.03,1.59) | 0.67 |
| 40-44 | 0.54 | (-0.33,1.42) | 0.45 | 0.58 | (-0.57,1.73) | 0.58 | 0.61 | (-0.87,2.08) | 0.75 | 0.68 | (-0.88,2.24) | 0.79 | 0.54 | (-0.54,1.61) | 0.55 |
| 45-49 | 0.79 | (0.10,1.48) | 0.35 | 0.97 | (0.07,1.87) | 0.46 | 0.48 | (-0.71,1.66) | 0.61 | 0.88 | (-0.35,2.12) | 0.63 | 0.75 | (-0.10,1.60) | 0.43 |
| 50-54 | 0.93 | (0.42,1.45) | 0.26 | 0.96 | (0.30,1.62) | 0.34 | 1.05 | (0.16,1.94) | 0.45 | 0.94 | (0.02,1.86) | 0.47 | 0.96 | (0.33,1.59) | 0.32 |
| 55-59 | 1.28 | (0.90,1.66) | 0.19 | 1.29 | (0.83,1.76) | 0.24 | 1.11 | (0.48,1.75) | 0.32 | 1.55 | (0.92,2.18) | 0.32 | 1.02 | (0.56,1.48) | 0.24 |
| 60-64 | 1.39 | (1.05,1.72) | 0.17 | 1.45 | (1.07,1.83) | 0.19 | 1.10 | (0.63,1.56) | 0.24 | 1.61 | (1.15,2.06) | 0.23 | 1.17 | (0.78,1.55) | 0.20 |
| 65-69 | 1.60 | (1.18,2.01) | 0.21 | 1.69 | (1.21,2.17) | 0.25 | 1.23 | (0.72,1.75) | 0.26 | 1.48 | (0.96,2.00) | 0.26 | 1.59 | (1.12,2.05) | 0.24 |
| 70-74 | 1.68 | (1.11,2.25) | 0.29 | 1.72 | (1.03,2.41) | 0.35 | 1.29 | (0.55,2.02) | 0.37 | 1.74 | (0.98,2.50) | 0.39 | 1.54 | (0.89,2.20) | 0.33 |
| 75-79 | 1.70 | (0.93,2.46) | 0.39 | 1.70 | (0.76,2.64) | 0.48 | 1.39 | (0.36,2.42) | 0.53 | 1.66 | (0.59,2.74) | 0.55 | 1.60 | (0.72,2.48) | 0.45 |
| 80-84 | 1.58 | (0.61,2.55) | 0.50 | 1.48 | (0.27,2.69) | 0.62 | 1.57 | (0.21,2.93) | 0.69 | 1.59 | (0.18,3.01) | 0.72 | 1.42 | (0.29,2.55) | 0.58 |
| **period** |  |  |  |  |  |  |  |  |  |  |  |  |  |  |  |
| 2005 | -0.32 | (-0.56,-0.09) | 0.12 | -0.48 | (-0.78,-0.19) | 0.15 | -0.03 | (-0.40,0.34) | 0.19 | -0.36 | (-0.74,0.02) | 0.20 | -0.33 | (-0.61,-0.05) | 0.14 |
| 2010 | -0.07 | (-0.15,0.01) | 0.04 | 0.02 | (-0.07,0.11) | 0.05 | -0.31 | (-0.46,-0.16) | 0.08 | -0.05 | (-0.18,0.08) | 0.06 | -0.07 | (-0.17,0.02) | 0.05 |
| 2015 | 0.40 | (0.18,0.62) | 0.11 | 0.47 | (0.18,0.75) | 0.14 | 0.34 | (0.00,0.68) | 0.17 | 0.41 | (0.05,0.77) | 0.18 | 0.40 | (0.14,0.67) | 0.13 |
| **cohort** |  |  |  |  |  |  |  |  |  |  |  |  |  |  |  |
| 1925-1929 | 2.73 | (1.64,3.81) | 0.56 | 3.03 | (1.62,4.44) | 0.72 | 1.85 | (0.02,3.69) | 0.94 | 2.99 | (1.19,4.78) | 0.92 | 2.56 | (1.22,3.91) | 0.69 |
| 1930-1934 | 1.89 | (1.02,2.76) | 0.44 | 2.12 | (1.00,3.25) | 0.57 | 0.87 | (-0.60,2.34) | 0.75 | 1.76 | (0.33,3.19) | 0.73 | 1.93 | (0.86,2.99) | 0.54 |
| 1935-1939 | 1.65 | (0.98,2.32) | 0.34 | 1.71 | (0.85,2.57) | 0.44 | 1.55 | (0.49,2.61) | 0.54 | 1.61 | (0.53,2.68) | 0.55 | 1.64 | (0.82,2.46) | 0.42 |
| 1940-1944 | 1.20 | (0.69,1.71) | 0.26 | 1.17 | (0.54,1.80) | 0.32 | 1.42 | (0.65,2.19) | 0.39 | 1.30 | (0.53,2.06) | 0.39 | 1.09 | (0.48,1.70) | 0.31 |
| 1945-1949 | 0.85 | (0.44,1.26) | 0.21 | 0.64 | (0.17,1.11) | 0.24 | 1.33 | (0.77,1.89) | 0.29 | 0.83 | (0.29,1.36) | 0.27 | 0.81 | (0.34,1.28) | 0.24 |
| 1950-1954 | 0.65 | (0.23,1.07) | 0.21 | 0.55 | (0.10,1.00) | 0.23 | 0.91 | (0.38,1.43) | 0.27 | 0.62 | (0.12,1.12) | 0.25 | 0.60 | (0.14,1.05) | 0.23 |
| 1955-1959 | 0.59 | (0.07,1.12) | 0.27 | 0.43 | (-0.15,1.01) | 0.30 | 0.89 | (0.22,1.56) | 0.34 | 0.42 | (-0.26,1.10) | 0.35 | 0.62 | (0.05,1.19) | 0.29 |
| 1960-1964 | 0.14 | (-0.55,0.83) | 0.35 | 0.00 | (-0.80,0.80) | 0.41 | 0.35 | (-0.58,1.28) | 0.47 | 0.05 | (-0.91,1.01) | 0.49 | 0.12 | (-0.64,0.89) | 0.39 |
| 1965-1969 | 0.32 | (-0.55,1.19) | 0.44 | 0.22 | (-0.81,1.25) | 0.53 | 0.20 | (-1.02,1.41) | 0.62 | 0.23 | (-1.05,1.50) | 0.65 | 0.22 | (-0.75,1.20) | 0.50 |
| 1970-1974 | -0.05 | (-1.11,1.02) | 0.54 | -0.28 | (-1.57,1.00) | 0.66 | 0.18 | (-1.33,1.69) | 0.77 | -0.38 | (-1.99,1.22) | 0.82 | -0.04 | (-1.25,1.16) | 0.62 |
| 1975-1979 | -0.07 | (-1.33,1.18) | 0.64 | -0.19 | (-1.73,1.35) | 0.78 | -0.13 | (-1.94,1.67) | 0.92 | -0.40 | (-2.33,1.52) | 0.98 | -0.10 | (-1.54,1.33) | 0.73 |
| 1980-1984 | -0.19 | (-1.64,1.26) | 0.74 | 0.02 | (-1.76,1.81) | 0.91 | -0.76 | (-2.88,1.36) | 1.08 | -0.33 | (-2.57,1.91) | 1.14 | -0.29 | (-1.95,1.38) | 0.85 |
| 1985-1989 | -0.33 | (-1.96,1.30) | 0.83 | -0.23 | (-2.25,1.80) | 1.03 | -0.52 | (-2.92,1.88) | 1.23 | -0.07 | (-2.59,2.45) | 1.29 | -0.52 | (-2.41,1.37) | 0.96 |
| 1990-1994 | 0.23 | (-1.53,1.99) | 0.90 | 0.14 | (-2.04,2.32) | 1.11 | -0.17 | (-2.74,2.40) | 1.31 | 0.21 | (-2.47,2.88) | 1.37 | 0.07 | (-1.97,2.11) | 1.04 |
| 1995-1999 | 0.70 | (-1.16,2.57) | 0.95 | -0.94 | (-3.63,1.76) | 1.37 | 1.69 | (-0.64,4.01) | 1.19 | 1.12 | (-1.42,3.67) | 1.30 | 0.19 | (-2.03,2.41) | 1.13 |
| 2000-2004 | 0.36 | (-1.59,2.3) | 0.99 | -0.11 | (-2.54,2.31) | 1.24 | 0.44 | (-1.89,2.78) | 1.19 | 0.33 | (-2.14,2.79) | 1.26 | -0.14 | (-2.46,2.19) | 1.19 |
| 2005-2009 | -2.42 | (-5.81,0.97) | 1.73 | -1.43 | (-5.32,2.46) | 1.98 | -2.12 | (-5.68,1.44) | 1.82 | -2.18 | (-5.73,1.37) | 1.81 | -1.48 | (-5.35,2.40) | 1.98 |
| 2010-2014 | -3.21 | (-8.12,1.70) | 2.51 | -2.74 | (-7.73,2.26) | 2.55 | -2.93 | (-7.66,1.81) | 2.41 | -3.15 | (-8.08,1.78) | 2.51 | -2.85 | (-7.71,2.01) | 2.48 |
| 2015-2019 | -5.03 | (-12.57,2.50) | 3.84 | -4.12 | (-11.71,3.47) | 3.87 | -5.04 | (-12.45,2.36) | 3.78 | -4.93 | (-12.45,2.60) | 3.84 | -4.42 | (-11.94,3.09) | 3.83 |

SE, standard error; 95% CI, 95% confidence interval

Figure S1. Sensitivity analysis of different random-walk orders for period effects in the BAPC model. Note: Red squares represent ASMR. The blue solid line indicates the model with a period effect using RW2, whereas the orange dashed line indicates the model with a period effect using RW1.

Figure S2. Sensitivity analysis of thyroid cancer ASMR predictions with different prediction horizons. Note: The blue line represents ASMR predictions up to 2025, whereas the orange line represents predictions up to 2030.
